# Relationship between Internet use and offline leisure activities among Chinese older adult people: a moderated mediation model

**DOI:** 10.3389/fpubh.2024.1458413

**Published:** 2025-01-08

**Authors:** Shuai Xiang, Qinwen Deng, Boli Chen

**Affiliations:** ^1^School of Public Policy and Management, Guangxi University, Nanning, China; ^2^School of Law, Chongqing University, Chongqing, China

**Keywords:** Internet use, offline leisure activities, environmental gerontology, active aging, older adults in China

## Abstract

**Background:**

The process of aging in Chinese society is accompanied by the concurrent development of the Internet. In recent years, the influence of Internet use on the activities of older adults has attracted growing interest and is now a significant focus of both public health and gerontological research. Nevertheless, there is a need for further empirical research in this area.

**Purpose:**

This study aimed to examine the mechanisms and interaction processes, as well as the relationship between Internet use and offline leisure activities among Chinese older adults. Theories of active aging and environmental gerontology guided the construction and assessment of the mediating role of self-perception of aging between Internet use and offline leisure activities. Additionally, the moderating role of community-based leisure places (CBLP) was examined.

**Methods:**

A moderated mediation model was constructed using the SPSS 27 PROCESS Macro. This study employs the most recent China Longitudinal Aging Social Survey (CLASS) 2020 cross-sectional data, and the valid sample comprises 8,180 Chinese older adult individuals aged 60 years or older.

**Results:**

(1) Internet use and offline leisure activities among older adults exhibited a significant positive correlation. (2) Self-perception of aging played a significant positive mediating role between Internet use and offline leisure activities. (3) CBLP significantly and positively moderated the relationship between Internet use and offline leisure activities.

**Conclusion:**

The Internet can be an effective tool for addressing the challenges of aging. Active Internet use may lead to more positive age perception and more active offline leisure activities, and efforts should be made to increase the digitization of the aging society. Positive self-perception of aging is an important psychological factor influencing the offline and online activities of older adults. It is imperative that the concept of active aging be vigorously promoted. Concurrently, community infrastructure serves as the foundation for the mutual promotion of online and offline activities. Attention should be given to the development of infrastructure for an age-friendly environment. These findings deepen the understanding of the consequences of interactions between online and offline activities, subjective mindsets, and objective environments for older adults.

## 1 Introduction

Population aging is a global phenomenon. The contraction of fertility and the increase in human longevity have inevitably led to a shift in the age structure of the population toward the older age groups (1). China’s society is aging at an alarming rate, making it the country with the largest older adult population in the world (2). As indicated by the data provided by China’s National Bureau of Statistics, in 2021, China will have a population over the age of 65, comprising 14.2% of the total population, thereby entering a period of profound demographic aging (3). The proportion of the population aged 65 and above is set to increase further, reaching 15.4% by 2022 (4). Due to its considerable population size, China is experiencing an unprecedented rate and scale of aging (5). The most conservative projections of the China Population Forecast Report 2023 indicate that the population over 65 in China is expected to reach 382 million by 2050, representing 24% of the global older adult population (6). By then, one out of every three Chinese will be over 65 years old. In this context, population aging has become a significant focus for the Chinese government. The Outline of the 14th Five-Year Plan (2021–2025) for National Economic and Social Development and Vision 2035 of the People’s Republic of China explicitly states that actively responding to population aging has been elevated to the status of a national strategy (7).

Each of us moves from childhood into middle age and then stumbles into old age. In the context of an aging global population, the physical and mental state, as well as the lifestyle intervention of older adults, represent a significant concern for scholars and policymakers engaged in the field of public health (8, 9). This study is specifically focused on these issues. At a time when technology is advancing rapidly, and the pace of society is accelerating, we are particularly concerned about whether the older adults are being isolated by the times or whether they can enjoy the benefits brought about by technological and social development. No other technology has had such a rapid impact on modern society as the Internet (10). Therefore, this paper intends to explore the impact of contemporary technology, represented by the Internet, on the psychological well-being and physical activity of older Chinese adults. Furthermore, an investigation of the role of community environments where older people congregate is essential to understanding the interaction between the Internet and older people’s physical and mental states.

The Internet represents an exceptional achievement of modern technology (10). The aging era is also the Internet era, and the aging of Chinese society is almost synchronized with information technology (11, 12). The advent of the Internet has transformed the way people live, and its influence on the older adults is multifaceted and complex. The impact of the Internet on older adults is one of the hot topics in gerontological research today (13–16). Internet use as an intervention strategy has become increasingly frequent in occupational therapy with older adults and has demonstrated positive effects on cognitive functioning and health status in older adults (17, 18). The accelerated integration of China’s middle-aged and older adult population into the Internet society has brought about profound changes in the structure and operation of the aging society (19). Discussing the impact of the Internet on older people in the context of digitization, the current and future focus of the field of active aging in China (20–22). It has been noted that there are significant differences in the use of the Internet by older persons in China, with some seniors being very familiar with using the Internet and others being disadvantaged by digital isolation (23, 24). In this paper, Chinese older adult people’s use of the Internet is used as an independent variable to explore its complex impact on real life.

Leisure is considered one of the main occupational activities of human beings, along with work and self-care (25, 26). As stated by scholars Lloyd and Auld in 2002, leisure activities are the most significant predictors of the quality of life for older adults (27). Leisure reduces the risk of declining health and losing independence in older adults through a preventive lifestyle-based occupational therapy intervention (28–30). In addition, Leisure plays a potential role in addressing disability, chronic illness, and mental illness (31). Occupational therapy applies leisure activities to the daily lives of older adults to maximize the improvement and restoration of their physical, psychological, and social interactions (31). By providing opportunities to fulfill life’s values and needs, leisure activities are essential to the long-term health and well-being of seniors (25). Therefore, “leisure activities” are often defined as voluntary activities that are undertaken for the purpose of enjoyment (32). Being involved in hobbies, the arts (such as dancing, chess, and poker), reading, watching TV, socializing, shopping, sports, exercising, volunteering, and participating in community, fitness, environmental, or library activities are all covered (32, 33). Leisure activities provide opportunities for people to fulfill their life values and needs. Through participation in leisure activities, people build social relationships, feel positive emotions, and gain additional skills and knowledge to improve their quality of life (34). Traditional leisure activities are mainly conducted in a physical, face-to-face manner. But as technology has changed the way people live, the Internet has also become a way for people to engage in leisure activities (35–38). To avoid confusion caused by overlapping concepts, this paper focuses on the impact of older adults’ online behavior on offline leisure (39, 40). A growing body of research has indicated that the Internet has significantly impacted the frequency, extent, and types of offline leisure activities engaged in by individuals of varying ages (40–42). There have been many similar studies focusing on young people (42–44). At a time when older people are rapidly becoming Internet users in large numbers, it is strategically important to conduct research on the impact of Internet use on offline leisure among older people (14, 45).

In their 1972 study, Lemon, Bengtson, and Peterson proposed a model of activity theory of aging, which posits that engaging in frequent and intimate activities is associated with a positive self-concept (46). A number of studies have shown that Internet use leads to the older adults being more receptive to new things and reducing the generation gap between the younger and middle-aged population, thus maintaining a more positive perception of aging (47–49). In occupational therapy, the Internet can increase solidarity among older adults, reduce isolation, and improve mental health (17). In the context of public health, self-perception of aging represents a significant psychological indicator of active aging (50). Scholars point out that seniors with a more positive perception are more likely to participate in social activities (50). Thus, self-perception of aging is a common mediating variable in gerontology-related studies (51–53). Consequently, this study used self-perception of aging as a mediating variable between Internet use and offline leisure activities among Chinese older adults.

The field of environmental gerontology puts forth a conceptual framework of “environmental support” (54). It is posited that environmental and individual factors interact and that human activity results from the interaction between individual capabilities and environmental resources (54, 55). The neighborhoods in which people live profoundly affect their daily lives, and previous research has shown that neighborhood environments play a significant role in moderating people’s physical and mental health (56, 57). After retirement, most of the activities of seniors are centered around their residences (58). Inclusive community environments increase community interaction and promote community integration of older persons (59). Community-based leisure places are a physical component of the community environment. The availability of community-based leisure places is crucial to older people’s leisure life (60, 61). Therefore, community-based leisure places, as a representation of the community environment, were the moderating variable in this study (62).

At present, there is a lack of targeted research on the relationship between Internet use and offline leisure activities among Chinese older adults. Therefore, this study aims to provide a more in-depth understanding of the relationship between Internet use and offline leisure activities among older adults while examining subjective psychological factors and objective environmental factors. Ultimately, our objective is to provide targeted recommendations for enhancing the quality of life of older individuals and facilitating active aging.

## 2 Conceptual framework and hypothesis raised

### 2.1 The Internet use and offline leisure activities

The key question is whether the Internet is good or bad for older people. Is the Internet a barrier for seniors or is it their key to the world (63)? The WHO policy recommendations on active aging identify information and communication technology (ICT) as an important tool for “lifelong learning and training” (8). Numerous studies have identified the Internet as a source of opportunity for older adults while affirming that the Internet has a positive impact on the well-being and successful aging of older adults (11, 64). Empirical studies of mental health confirm that the Internet significantly reduces loneliness and improves the subjective well-being of older adults (13, 14, 65, 66). In improving social interaction among older people, the Internet also plays a positive role (63). A study suggests that online social media, as represented by Facebook, plays a useful role in facilitating interaction with the outside world for the solitary older adults and significantly improves their quality of life (14). Clinical studies have concluded that online brain training programs can have a positive impact on improving cognitive function (67, 68). Scholars in the field of occupational therapy have identified a correlation between the use of email and computers by older adults and a number of beneficial factors, including younger attitudes, higher levels of education, and more positive behavioral coping styles (69). This observation has led to the recommendation of computer-based activity interventions in older adult communities (69).

Previous studies on “silver-surfers” (70, 71) have mostly focused on the impact of the Internet on social interaction, social participation, and social support of the older adults, but less attention has been paid to the relationship between the Internet and offline leisure activities. A study conducted in Finland indicates that the influence of Internet usage on offline leisure activities is increasing as ICT becomes more significant in individuals’ daily routines (35, 40). Meanwhile, a survey among Chinese seniors aged over 60 shows that the most common purposes of Internet use are socializing, participating in group activities, entertainment, and travel (21). The Internet serves both their online and offline lives, which is consistent with our observations. Using square dance as an example, this activity is popular among middle-aged and older adult women in China. Short videos, WeChat, and other online social platforms allow seniors to easily connect with others in their community who share an interest in square dancing and form a regular team (45). From this example, it is evident that online socialization and offline leisure activities mutually reinforce each other. Thus, we argue that the Internet plays a significant and positive correlation role in the offline leisure activities of older adults.

*Hypothesis* 1: There is a positive correlation between Internet use and offline leisure activities among Chinese older adults.

### 2.2 The mediating role of self-perception of aging

Perception of aging can be defined as beliefs about the experience of aging, attitudes, and emotional reactions to the aging process (72). The capacity to reflect on and explain the aging process is a unique attribute of the human condition that can influence the aging process itself (73). Activity Theory of Aging (ATA) provides a foundation for understanding the relationships among Internet use, self-perception of aging, and offline leisure activities. This theory suggests that older people have a natural tendency to seek out interactions with others and participate in groups and social events (74). Positive self-perception of aging indicates that older individuals have a desire to maintain social connections, a sense of continuing purpose, and the ability to be independently active (75). Technology, represented by the Internet, influences self-perception of aging (49). Most studies have concluded that the Internet is significantly and positively associated with positive self-perception of aging in older adults (49, 63). Older people who use the Internet are mentally younger, have better physical and mental health, are more socially engaged, and have a greater sense of well-being and higher quality of life than those who do not use the Internet (20, 49, 63).

The Internet can influence self-perception of aging, and seniors with a positive self-perception of aging tend to be more socially active and engage in more offline leisure activities. Therefore, in this study, self-perception of aging was used as a mediator between Internet use and offline leisure activities among older adults.

*Hypothesis* 2: There is a positive correlation between Internet use and self-perception of aging among older adults.

*Hypothesis* 3: There is a positive correlation between self-perception of aging and offline leisure activities among older adults.

*Hypothesis* 4: Self-perception of aging is a mediator between Internet use and offline leisure activities among older adults.

### 2.3 The moderate role of community-based leisure places

Community-based leisure places are recreational areas and buildings open to the public. Examples of community-based leisure places include parks, green spaces, churches, gymnasiums, boardrooms, and plazas. Providing venues for exercise, recreation, and social interaction, community-based leisure places play an important role in the physical and mental health of older adults (61, 76). Aging will lead to a decrease in the mobility of older adults, whose daily activities are primarily centered around their residences in the community (58). Environmental Gerontology recognizes that the community is the terminal for serving the needs of older people and directly determines the effectiveness of active aging policies (77). The concept of age-friendly communities has been proposed to promote the health and welfare of older persons and encourage their participation and interaction (61, 62). Community-based leisure places play a crucial role in creating age-friendly communities. A study of geriatric behavior in Poland found that the absence of offline leisure activities was linked to the lack of recreational facilities and public spaces (78). Environmental factors, such as community-based leisure places, significantly affect older individuals’ desire to participate in offline leisure activities. Community-based leisure places provide important physical conditions for seniors to extend their social interactions online into their offline lives (61). A safe and comfortable environment will lead to a greater willingness to engage in community activities (55, 79). Therefore, this study argues that community-based leisure places play a moderating role in self-perception of aging and offline leisure activities, as well as Internet use and offline leisure activities.

*Hypothesis* 5: Community-based leisure places positively moderate the relationship between older adults’ Internet use and offline leisure activities.

*Hypothesis* 6: Community-based leisure places positively moderate the relationship between older adults’ self-perception of aging and offline leisure activities.

**Figure 1:**
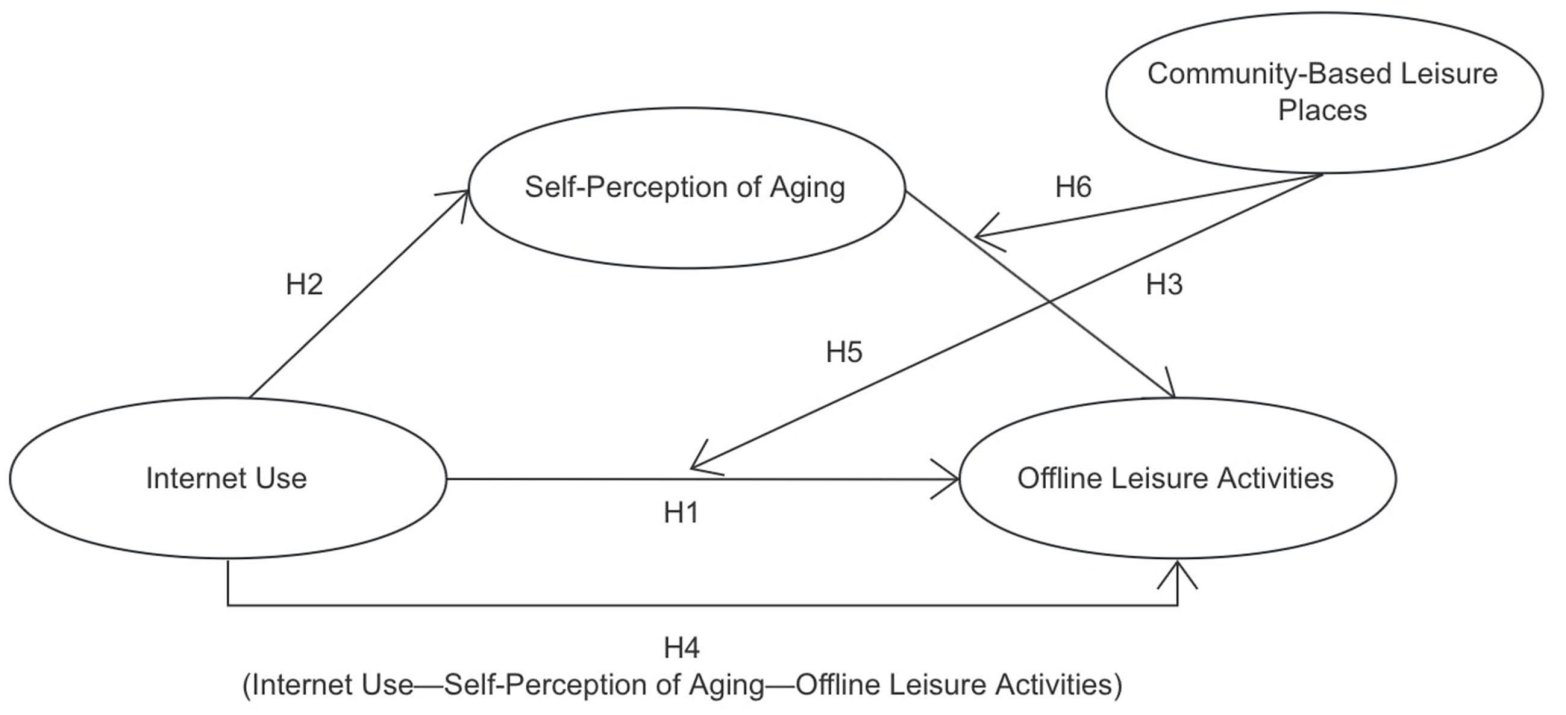
Conceptual framework.

However, there is a lack of research exploring the relationship between Internet use and offline leisure activities among Chinese older adults, as well as targeted studies using self-perception of aging as a mediating variable and community-based leisure places as a moderating variable. To explore these issues further, Figure 1 illustrates the relationships identified in this study.

This study contributes in three parts. Firstly, we examine the relationship between Internet use and offline leisure activities among older adults in the Chinese context. Secondly, based on ATA, this study focuses on the mediating role of self-perception of aging between Internet use and offline leisure activities. Finally, it aims to examine the moderating role of community-based leisure places between Chinese older people’s Internet use and offline leisure activities, as well as self-perception of aging and offline leisure activities, from Environmental Gerontology’s perspective.

## 3 Materials and methods

This section describes the data collection, survey methodology, sample, key variables, and methods used to analyze the data.

### 3.1 Data

This paper uses the most recently published 2020 cross-sectional data from China Longitudinal Aging Social Survey (CLASS)^1^. CLASS is a nationwide tracking social survey designed by the Institute of Gerontology of Renmin University of China and implemented by the China Survey and Data Center of Renmin University of China. CLASS started two trial surveys in 2011 and 2012, respectively. In the summer of 2014, CLASS initiated a nationwide baseline survey, followed by three subsequent follow-up surveys of baseline respondents conducted in 2016, 2018, and 2020. The baseline survey of 2014 spanned 10 months. The survey covered 29 provincial administrative regions in mainland China. CLASS 2020 was conducted among individuals aged 60 years and older.^2^ The survey covers various aspects, including the socio-economic situation, health and related services, retirement planning, social support, as well as physical and mental health.

In accordance with the Statistics Law of the People’s Republic of China, CALSS uses probability sampling, which minimizes the influence of bias and subjective factors. Probability sampling ensures that each aggregate unit in the target aggregate has a known probability of being drawn and that the structure of the constituent samples is consistent with the structure of the aggregate (80). Probability sampling markedly enhances the representativeness and accuracy of the sample and avoids endogeneity problems caused by sample selection bias in the sampling section. CLASS employs a multi-stage probability sampling method, with the county-level units serving as the Primary Sampling Units (PSUs), the village councils in rural areas/communities, and neighborhood councils in urban areas serving as the Secondary Sampling Units (SSUs). Firstly, PSUs were randomly selected from a sampling frame containing all county-level units using the sampling “proportional to population size.” Secondly, SSUs were selected from PSUs in accordance with the same methodology. Finally, individuals aged 60 and above were selected randomly from each SSU based on a sampling map. The baseline sample for CLASS 2014 encompassed 134 counties and districts across mainland China, comprising a total of 462 villages and residential committees. The sample included 11,511 individuals aged 60 years or above.

The total sample size for the CLASS 2020 survey is 11,398 individuals. To exclude the interference of other factors, the inclusion criteria of the data samples in this study were: “agree to be interviewed.” The exclusion criteria were: (1) Values of “Do not know” or “Refused to answer” for the required variables and (2) missing values for the required variables. By processing the initial sample data as described above, 8,180 valid samples were included in this study. The data was analyzed by SPSS 27 software. Samples with missing values have been excluded from the analysis in this study.

CLASS was conducted in accordance with the ethical principles of the Declaration of Helsinki. We received authorization to use the publicly accessible CLASS 2020, which is available.^3^ This study was conducted based on de-identified publicly available CLASS 2020 data.

### 3.2 Variables selection

This section describes the key variables and control variables.

#### 3.2.1 Independent variable

Internet use was selected as the independent variable in this study. Question D18 from the CLASS2020 questionnaire was selected: “How often do you surf the Internet? (Including the use of mobile electronic devices such as cell phones to surf the Internet)” D18 is a single-selection question that is answered on a 5-point Likert scale type: “Never surf the Internet = 1, surf the Internet annually = 2, surf the Internet monthly = 3, surf the Internet weekly = 4, surf the Internet daily = 5.” The score obtained is directly proportional to the frequency of Internet use.

#### 3.2.2 Dependent variable

The frequency of participation in offline leisure activities was selected as the dependent variable in this study. Question D15-2 was selected, which consists of six sub-questions in total: “In the previous year, how frequently did you engage in the following activities: (1) Religious activities; (2) Attending universities for the aged or training courses; (3) Watching TV/listening to the radio/reading books/reading newspapers/listening to operas; (4) Singing/playing musical instruments; (5) Playing mah-jongg/chess/cards, etc.; and (6) Square dancing (Excluding online participation)”.

The answers to the six sets of questions are recoded in a harmonized manner: “never participate = 0, several times a year = 1, at least once a month = 2, at least once a week = 3, almost every day = 4.” The question was answered on a 5-point Likert scale type. The higher the score, the more frequently the older adult participate in leisure activities. Add up the scores from the six sets of questions and divide the sum by six. The final score is indicative of the level of engagement with offline leisure activities.

#### 3.2.3 Mediating variable

Self-perception of aging was selected as the mediating variable in this study. Self-perception of aging is an individual’s subjective evaluation of aging, which is one of the reliable indicators of an individual’s aging mental status. Question E6 from the CLASS 2020 questionnaire was selected, which consists of eight sub-questions in total: “Do you feel that the following description fits your current reality? (1) I am willing to participate in some of the work of the village committee/ urban residents committee; (2) I always have frequently desired to contribute to the community; (3) I still love learning; (4) I believe that I am still a valuable person for the society; (5) Society is changing so rapidly that it is difficult to adapt to such changes; (6) More and more of today’s new ideas are difficult for me to accept; (7) More and more of today’s social policies are difficult for me to accept; (8) Social changes are now increasingly unfavorable to the older adults.” In CLASS 2014, E6 is classified within the “Aging Perception” subsection of the E section. In CLASS 2020, the subordination and content of E6 remain unchanged. In consideration of the historical subordination and content of E6, we employ E6 to indicate the self-perception of aging.

For questions (1) to (4), this study utilized the original scoring rule: “I completely agree = 5; I mostly agree = 4; I half agree = 3; I mostly disagree = 2; I completely disagree = 1.” We recode question (5) to (8): “I completely disagree = 5; I mostly disagree = 4; I half agree = 3; I mostly agree = 2; I completely agree = 1.” As a result, questions (1)–(8) were answered with the same values. The higher the score, the more positive the senior’s self-perception of aging is. Add up the scores from the eight sets of questions and divide the sum by eight. The final score represents self-perception of aging.

#### 3.2.4 Moderating variable

Community-based leisure places were selected as the moderating variable. Question D11 from the CLASS 2020 questionnaire was selected: “Are any of the following community-based leisure places available in your community: (1) activity room for the older adults; (2) fitness place/facility; (4) chess/mahjong room; (4) library; (5) outdoor activity area; (6) other (please specify); (7) none of the above (multiple-choice).” D11 is a multiple-choice question. The score increases with the variety of places available for leisure activities in the community. Score 0 for option (7).

#### 3.2.5 Control variables

Based on the published relevant studies, this study concludes that gender, education level, ethnic group, spouse, solitary, household registration, geographic division, self-assessed economic situation, and self-assessed health situation are the significant factors affecting older peoples’ leisure activities (41, 81, 82). Therefore, these types of variables will be used as control variables in this study. The value 1 is assigned to males and the value 2 is assigned to females. The education level of “illiterate, primary education, secondary education, higher education” is assigned a value of 1, 2, 3, and 4 in that order. Han nationality is assigned a value of 1, and minority nationality is assigned a value of 2. If an individual has a spouse, they will be assigned a value of 1. If they do not have a spouse, they will be assigned a value of 0. Solitary is assigned a value of 0, not solitary is assigned a value of 1. Rural registration is assigned a value of 1, and urban registration is assigned a value of 2. East is assigned a value of 1, central is assigned a value of 2, and west is assigned a value of 3.^4^ The self-assessed economic situation of “poor, average, good,” is assigned a value of 1, 2, and 3 in that order. The self-assessed health situation is assigned a value of “1, 2, 3, 4, 5,” the higher the value, the better the self-assessed health status.

### 3.3 Methods

This study began with a description of four sets of key variables and examined the correlations between four sets of key variables. Subsequently, this paper adopted Structural Equation Modeling (SEM) as an analytical technique. The study investigated the correlation between Internet use and offline leisure activities in three phases (H1). It also explored the mediating effect of self-perception of aging and the moderating role of community-based leisure places. We examined the mediation model and the moderated mediation model through two types of analyses, as suggested by Hayes (83). The SPSS PROCESS Macro model 4 was used to test the mediating aspect (H2, H3, and H4). The SPSS PROCESS Macro model 15 was then used to incorporate the moderating effects into the full model (H5 and H6) (83). Model 4 and Model 15 passed the special bootstrapping test with 95% confidence intervals (the number of bootstrap samples is 5,000) with important findings.

## 4 Results

### 4.1 Descriptive

Table 1 presents the socio-demographic characteristics of the respondents. As unordered categorical features, control variables were described by frequencies. Table 1 displays a gender-balanced sample with slightly more males (*N* = 4,171, 51.5%), which is consistent with China’s total gender ratio (84). The regional division is consistent with China’s population distribution, with the largest population residing in the eastern region (*N* = 3,385, 41.4%), followed by the central region (*N* = 2,923, 35.7%), and the smallest population in the western region (*N* = 1872, 22.9%) (85). The registration of households is balance distributed between urban (*N* = 3,750, 45.8%) and rural areas (*N* = 4,430, 54.2%), with slightly more respondents residing in rural areas. One-tenth of the total number of respondents were older adult people living alone (*N* = 827, 10.1%), while one-quarter of the total number of respondents were seniors without spouses (*N* = 2071, 25.3%). It is important to pay attention to their physical and mental health. The respondents are predominantly Han Chinese, comprising 94.3% (*N* = 7,713) of the total number, which is slightly higher than the percentage of Han Chinese in the national population (85). The education levels of the older adult respondents were concentrated at the primary (*N* = 3,376, 41.3%) and secondary (*N* = 2,788, 34.1%) levels, followed by illiterate (*N* = 1991, 24.3%). The proportion of older adult respondents with higher education was the lowest among all levels (*N* = 25, 0.3%). A majority of the older adult respondents rated their financial situation as average (*N* = 5,831, 71.3%). Approximately 40% of respondents reported relatively good health (*N* = 3,285, 40.2%). The sample for this study is consistent with the 7th Population Census of China and recent demographic information released by the National Bureau of Statistics of China. Therefore, the sample of this research can reflect the overall situation in China.

**Table 1:** Sociodemographic characteristics of the participants and descriptive statistics for main variables.

Dimension	Variable	Indicators	N	PCT(%)	Mode
Control Variables	Gender	Male	4,171	51.5%	Male
		Female	4,009	49.0%	
	Geographic division	East	3,385	41.4	East
		Central	2,923	35.7	
		West	1872	22.9	
	Household registration	Rural	4,430	54.2	Rural
		Urban	3,750	45.8	
	Solitary	Solitary	827	10.1	Non-solitary
		Not solitary	7,353	89.9	
	Spouse	Have spouse	6,109	74.7	Have spouse
		Have not	2071	25.3	
	Ethnic group	Han-nationality	7,713	94.3	Han nationality
		Minority-nationality	467	5.7	
	Education level	Illiterate	1991	24.3	Primary
		Primary	3,376	41.3	
		Secondary	2,788	34.1	
		Higher	25	0.3	
	Self-assessed economic situation	Poor	913	11.2	Average
		Average	5,831	71.3	
		Good	1,436	17.6	
		Excellent	189	2.3	
	Self-assessed health situation	Poor	1,038	12.7	Good
		Average	3,062	37.4	
		Good	3,285	40.2	
		Excellent	606	7.4	
Dimension	Variable	Mean	Std. dev	Min	Max
Independent	IU	1.9222	1.6148	1	5
Dependent	OLA	0.7862	0.5780	0	4
Mediator	SPA	3.0102	0.5154	1	5
Moderator	CBLP	1.6408	1.4978	0	5

*N = 8,180. IU, Internet Use; OLA, Offline Leisure Activities; SPA, Self-Perception of Aging; CBLP, Community-Based Leisure Places.*

**Figure 2:**
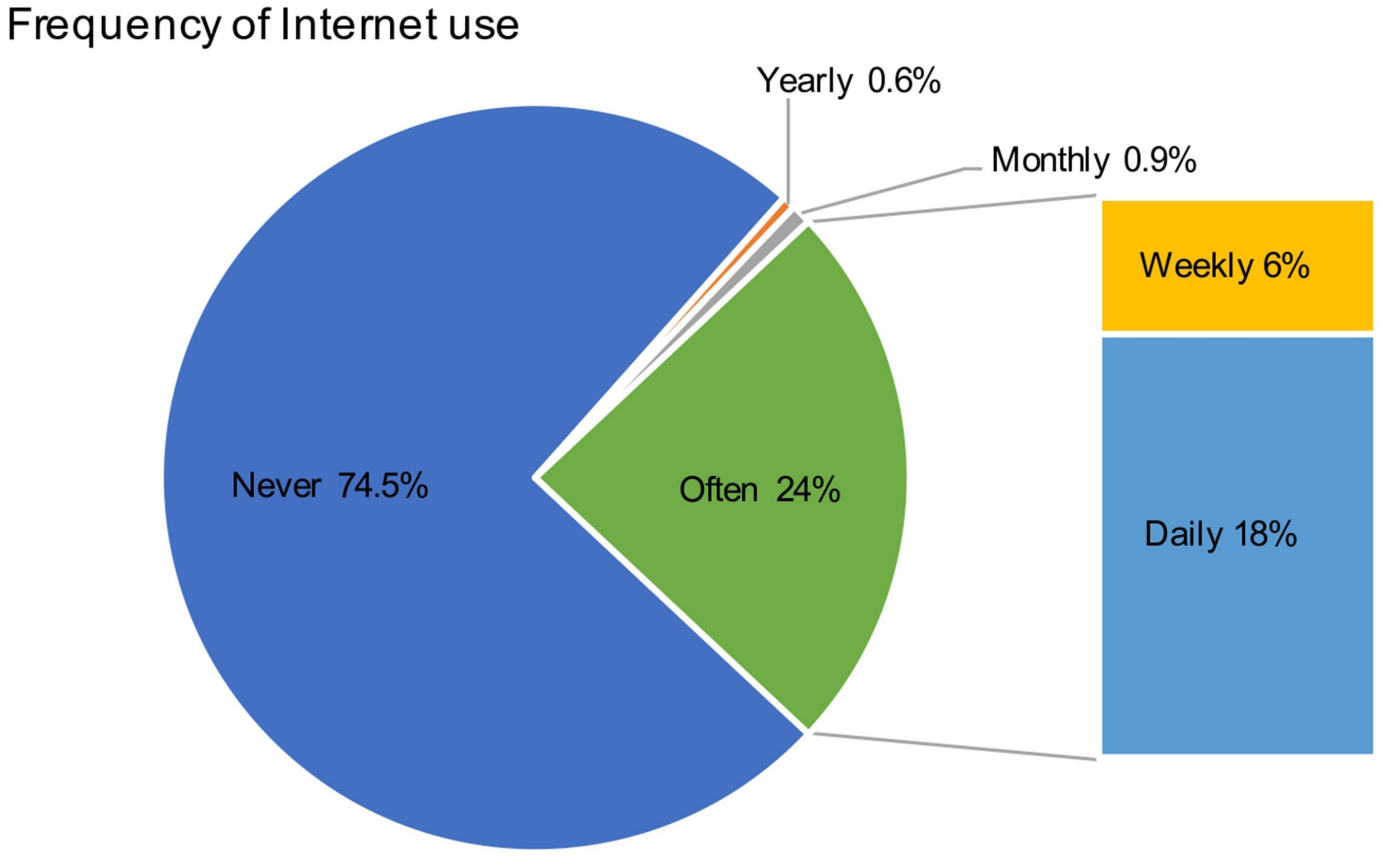
Internet use among Chinese older adults respondents.

The key variables are described in Table 1. The mean score for Internet use among respondents was 1.9222 (SD = 1.6148). The description of the frequency of the independent variables indicated a polarization in the use of the Internet by the respondents (Figure 2). A total of 24% (*N* = 1,473) of respondents indicated that they utilize the Internet on a daily or weekly basis. This group of older adults can use the Internet proficiently and frequently, and the Internet has become a part of their lives. A mere 1.5% of seniors were found to utilize the Internet on a monthly (*N* = 72, 0.9%) or annually (*N* = 47, 0.6%) basis. This is a very small percentage of seniors who can be identified as having no Internet usage habits. Furthermore, it can be observed that approximately three-quarters (*N* = 6,101, 74.5%) of respondents do not utilize the Internet. As illustrated in Table 1, the mean score of older adult respondents’ participation in offline leisure activities was 0.7862 (SD = 0.578), and most older adult respondents are in the habit of performing leisure activities. The mean score for the older adult respondents on the self-perception of aging was 3.0102 (SD = 0.5154), indicating that the majority of the older adults exhibited a calm and tranquil subjective mindset. The mean score of CBLP was 1.6408 (SD = 1.4978), indicating that the majority of neighborhoods where respondents reside offer a minimum of one or two types of recreational leisure places.

### 4.2 Correlation coefficients

Pearson correlation analysis and frequency description were used to examine the relationship between Internet use, offline leisure activities, self-perception of aging, and CBLP. The results are presented below:

Table 2 above shows that every two key variables were significantly and positively correlated (correlation coefficient > 0, *p* < 0.05). Specifically, Internet use was significantly and positively associated with offline leisure activities (*β* = 0.337, *p* < 0.01). Internet use was significantly and positively associated with self-perception aging (*β* = 0.126, *p* < 0.01). Internet use was significantly and positively associated with CBLP (*β* = 0.137, *p* < 0.01). Self-perception of aging was significantly and positively associated with offline leisure activities (*β* = 0.087, *p* < 0.01). Self-perception of aging was significantly and positively associated with CBLP (*β* = 0129, *p* < 0.01). CBLP was significantly and positively associated with offline leisure activities (*β* = 0.258, *p* < 0.01).

**Table 2:** Intercorrelation statistics of the main variables of the study participants.

Variable	1	2	3	4
1. IU	—			
2. OLA	0.337^**^	—		
3. SPA	0.126^**^	0.087^**^	—	
4. CBLP	0.137^**^	0.258^**^	0.129^**^	—

*N* = 8,180. IU, Internet Use; OLA, Offline Leisure Activities; SPA, Self-Perception of Aging; CBLP, Community-Based Leisure Places. ^*^p < 0.05. ^**^p < 0.01. ^***^p < 0.001.

### 4.3 Hypothesis testing

#### 4.3.1 The hierarchical regression and mediator test

We applied Model 4 of SPSS Process Marco 3.2 to test H1, H2, H3, and H4. During the SEM analysis, Internet use, offline leisure activities, and self-perception of aging were assessed as first-order constructs. Subsequently, the role of the self-perception of aging as a mediator was subjected to a second-order evaluation. Table 3 shows the hierarchical regression analysis results and Table 4 shows the test of mediating effect.

**Table 3:** Results of chain mediating effect.

Hypothesis	Structural path		Standardized estimate(β)	Standard error	t-value
H1	IU → OLA		0.0804	0.0039	20.4184^***^
H2	IU → SPA		0.0274	0.0038	7.1403^***^
H3	SPA→OLA		0.0384	0.0113	3.3908^***^
Control Variables	1. Gender	Male→OLA	−0.0569	0.0119	−0.47926
	2. Education Level	Primary education→OLA	0.0688	0.0150	4.5881^***^
		Secondary education→OLA	0.0880	0.0172	5.1223^***^
		Higher education→OLA	−0.1873	0.1085	−1.7266
	3. Ethnic group	Han → OLA	0.1655	0.0253	6.5304^***^
	4. Spouse	Have spouse→OLA	0.0342	0.0158	2.1638^*^
	5. Solitary	Solitary→OLA	−0.0354	0.0221	−1.6017
	6. Household registration	Rural→ OLA	−0.0960	0.0129	−7.4358^***^
	7. Geographic division	East→ OLA	0.2764	0.0160	17.3029^***^
		Central→OLA	0.0408	0.0156	2.6146^***^
	8. Self-assessed economic situation		0.0289	0.0108	2.6722^**^
	9. Self-assessed health situation		0.0402	0.0067	5.9849^***^
*F* = 153.1375^***^					
R^2^ = 0.196					

*N* = 8,180. Virtual variables are Female (Gender), Illiterate (Education level), Minority (Ethnic group), Have no spouse (Spouse), Non-solitary (Solitary), Urban (Household Registration), and West (Geographic division). IU, Internet Use; OLA, Offline Leisure Activities; SPA, Self-Perception of Aging; CBLP, Community-Based Leisure Places. ^*^p < 0.05. ^**^p < 0.01. ^***^p < 0.001.

**Table 4:** The mediating role of SPA in the relationship between IU and OLA.

	Effect	Boot SE	Boot LLCI	Bott ULCI	Relative Effect
Total effect	0.0815	0.0039	0.0738	0.0892	100%
Direct effect	0.0804	0.0039	0.0727	0.0882	98.65%
Indirect Effect	0.0011	0.0003	0.0004	0.0018	1.35%

Boot, Bootstrap; SE, Standard Error; LLCI, Lower Limit of Confidence Interval; ULCI, Upper Limit of Confidence Interval; IU, Internet Use; OLA, Offline Leisure Activities; SPA, Self-Perception of Aging.

Table 3 shows that Internet use significantly and positively relates to self-perception of aging (*β* = 0.0274, *p* < 0.0001) and offline leisure activities (*β* = 0.0805, *p* < 0.0001), thereby supporting H1 and H2. Furthermore, we found that self-perception of aging was positively and significantly associated with offline leisure activities (*β* = 0.0384, *p* < 0.0001), thus supporting H3.

In Table 4, after controlling for nine sets of demographic information, the direct effect of Internet use on offline leisure activities accounts for 98.65% of the total effect, with a 95% confidence interval of [0.0727, 0.0882] and 0 was excluded, indicating that the direct effect is significant and H1 is supported. The indirect effect of Internet use on offline leisure activities through self-perception of aging accounted for 1.35% of the total effect, with a 95% confidence interval of [0.0004, 0.0018] and 0 was excluded, indicating that the mediating effect of self-perception of aging is significant and H4 is supported. This study belongs to the sociology of aging and behavioral Sciences (86). In the social and behavioral sciences, the coefficients and effect sizes observed in causal mediation analyses are typically small, particularly when the sample size is large (87). This finding aligns with H4. The mediating effect share and coefficient of H4 are relatively minor, yet the result is still statistically significant, indicating that the mediating effect of self-perception of aging is a worthy area for investigation (87).

#### 4.3.2 Test of the moderation effect of community-based leisure places

In this study, Model 15 of SPSS Process Marco 3.2 was used to test the moderating effect of CBLP, control for nine groups of demographic information, standardize the predictive variables, and test the moderated mediation model.

**Figure 3:**
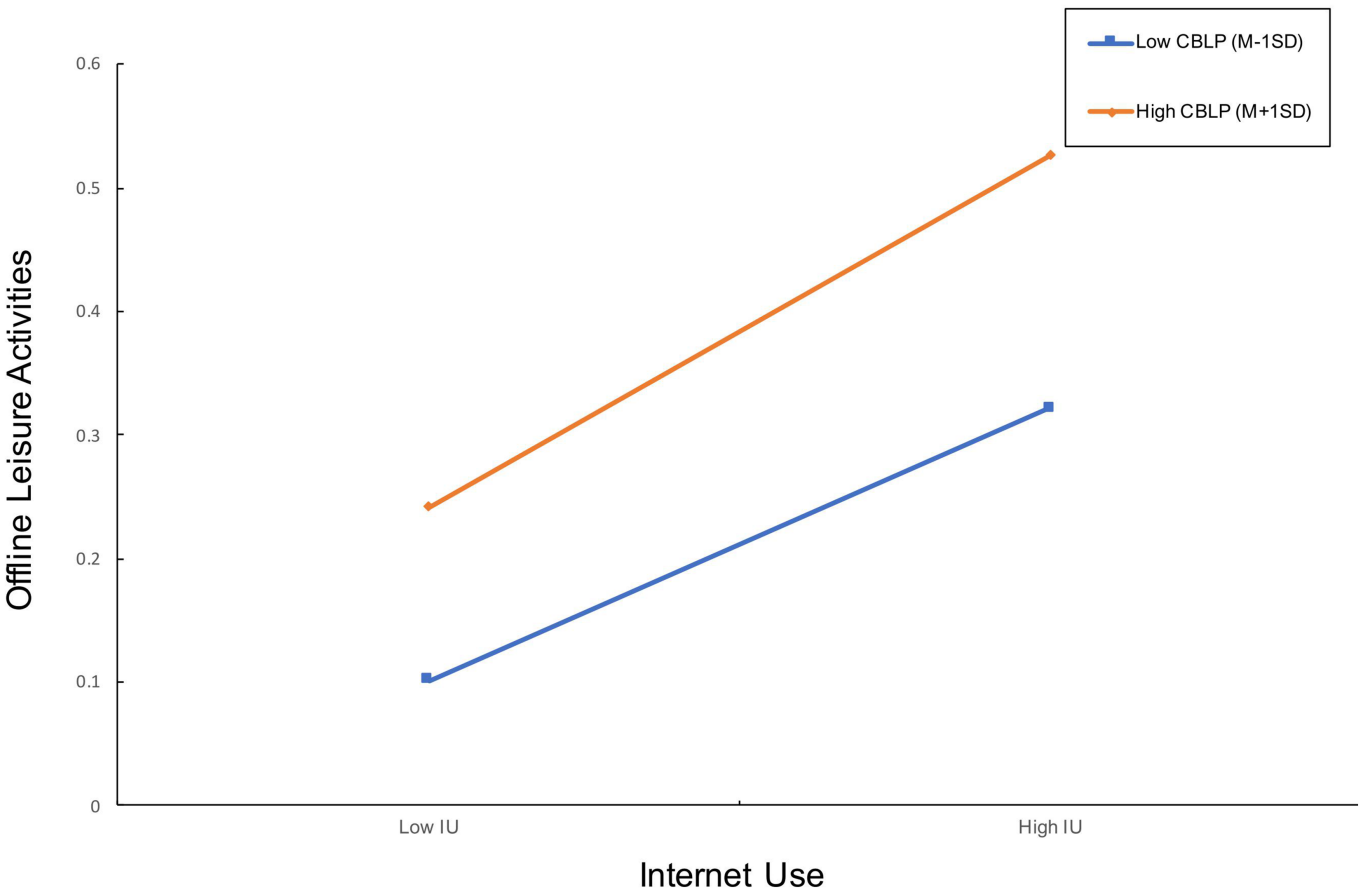
The interaction plot for H5.

As shown in Table 5, the moderation effect of community-based leisure places on Internet use and offline leisure activities is significant at low, average, and high levels. According to the slope test results given in Table 6 and the interaction plot given in Figure 3, the interaction effect is stronger in the case of a high level of community-based leisure places (Effect = 0.0883; SE = 0.0048; *p* < 0.0001) than in the case of a low level of community-based leisure places (Effect = 0.0682; SE = 0.0056; *p* < 0.0001). Therefore, we concluded that H5 is supported.

**Table 5:** The moderated mediation model with SPA as mediator and CBLP as moderator.

Variable		SPA			OLA		
		Model 1			Model 2		
		B	SE	t-value	B	SE	t-value
Gender	Male	0.0152	0.0116	1.3104	−0.0522	0.0117	−4.4585^***^
Education level	Primary	−0.0078	0.0146	−0.5310	0.0548	0.0149	3.6868^***^
	Secondary	0.0729	0.0168	4.3473^***^	0.0708	0.0170	4.1610^***^
	Higher	0.0934	0.1058	0.8829	−0.1914	0.1071	−1.7872
Ethnic group	Han	0.0495	0.0247	2.0023^*^	0.1704	0.0250	6.8053^***^
Spouse	Have spouse	−0.0024	0.0154	−0.1575	0.0387	0.0156	2.4763
Solitary	Solitary	0.0005	0.0216	0.0249	−0.0188	0.0219	−0.8577
Household registration	Rural	0.0003	0.0126	0.0249	−0.0545	0.0131	−4.1524^***^
Geographic division	East	−0.0222	0.0156	−1.4271	0.2479	0.0159	15.5444^***^
	Central	−0.0300	0.0152	−1.9684	0.0381	0.0154	2.4655^*^
Self-assessed economic situation		0.0703	0.0065	10.7938^***^	0.0420	0.0066	6.3286^***^
Self-assessed health situation		0.0276	0.0105	2.6173^**^	0.0254	0.0107	2.3755^**^
IU		0.0274	0.0038	7.1414^***^	0.0782	0.0039	19.8694^***^
SPA		/ / /			0.0206	0.0113	1.8193
CBLP		/ / /			0.0577	0.0042	13.8598^***^
IU × CBLP		/ / /			0.0067	0.0023	2.8975^**^
SPA×CBLP		/ / /			−0.0111	0.0075	−1.4849
		*F* = 24.6630^***^			*F* = 133.0600^***^		
		R^2^ = 0.0378			R^2^ = 0.2170		

*N =* 8,180. Virtual variables are Female (Gender), Illiterate (Education level), Minority (Ethnic group), Have no spouse (Spouse), Non-solitary (Solitary), Urban (Household Registration), and West (Geographic division). IU, Internet Use; OLA, Offline Leisure Activities; SPA, Self-Perception of Aging; CBLP, Community-Based Leisure Places. ^*^p < 0.05. ^**^p < 0.01. ^***^p < 0.001.

**Table 6:** CBLP as a moderator between IU and OLA.

CBLP	Effect	SE	t-value	*p*-value	LLCI	ULCI
Low CBLP	0.0682	0.0056	12.1493	<0.0001	0.0572	0.0792
Average CBLP	0.0782	0.0039	19.8694	<0.0001	0.0705	0.0860
High CBLP	0.0883	0.0048	18.2491	<0.0001	0.0788	0.0977

SE, Standard Error; LLCI, Lower Limit of Confidence Interval; ULCI, Upper Limit of Confidence Interval; IU, Internet Use; OLA, Offline Leisure Activities; CBLP, Community-Based Leisure Places.

**Figure 4:**
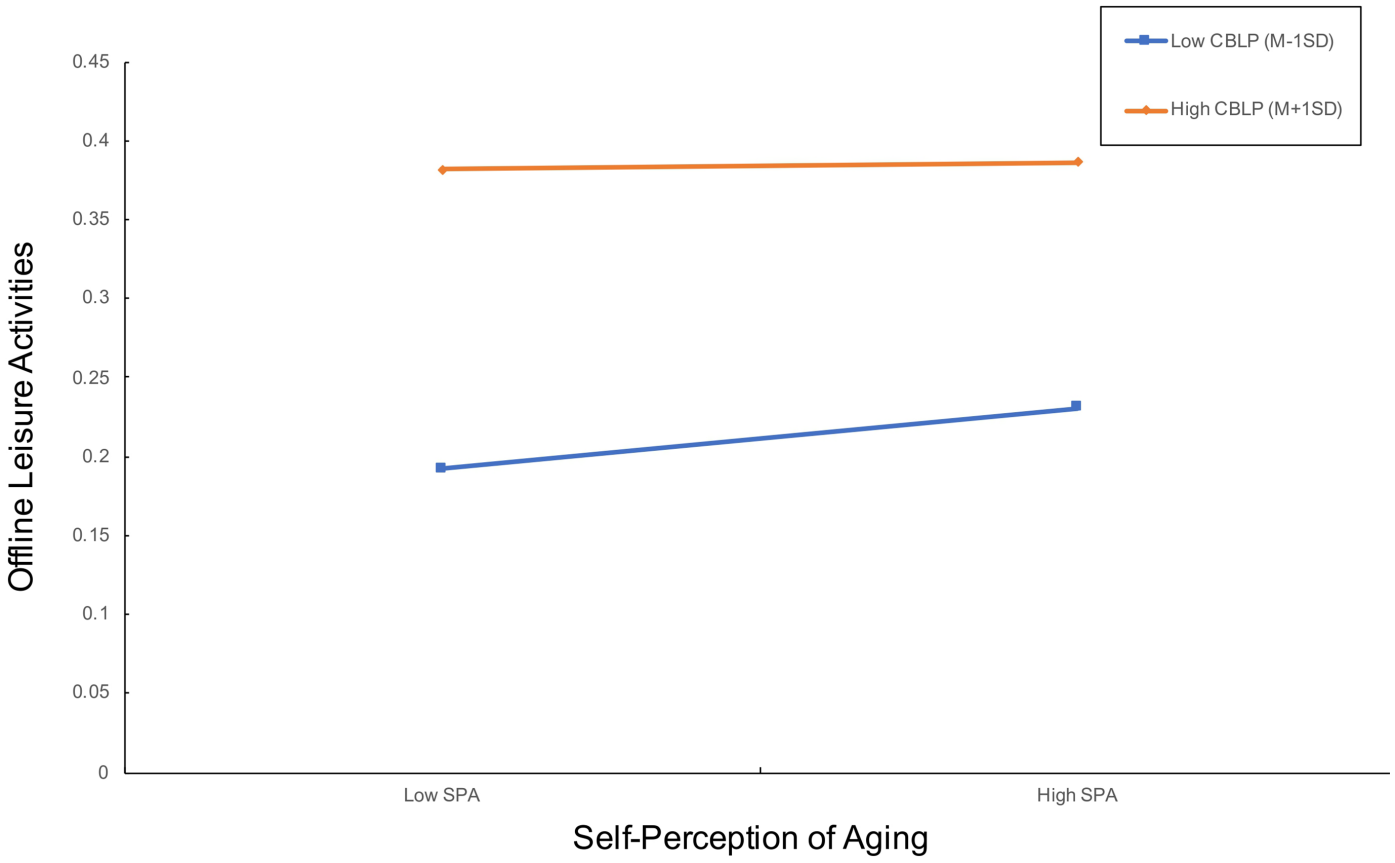
The interaction plot for H6.

However, there was no support for the moderator effect of community-based leisure places on the relationship between self-perception and offline leisure activities, as seen under Model 2 in Table 5. Furthermore, according to the slope test results presented in Table 7 and the interaction plot shown in Figure 4, the positive correlation between self-perception of aging and offline leisure activities does not differ in the cases of average and high levels of community-based places. Therefore, H6 is not supported.

**Table 7:** CBLP as a moderator between SPA and OLA.

CBLP	Effect	SE	t-value	*p*-value	LLCI	ULCI
Low CBLP	0.0373	0.0167	2.2381	0.0252	0.0046	0.0699
Average CBLP	0.0206	0.0113	1.8193	0.0689	−0.0016	0.0428
High CBLP	0.0039	0.0152	0.2589	0.7958	−0.0259	0.0337

SE, Standard Error; LLCI, Lower Limit of Confidence Interval; ULCI, Upper Limit of Confidence Interval; SPA, Self-Perception of Aging; OLA, Offline Leisure Activities; CBLP, Community-Based Leisure Places.

### 4.4 The results of moderated mediation model

**Figure 5:**
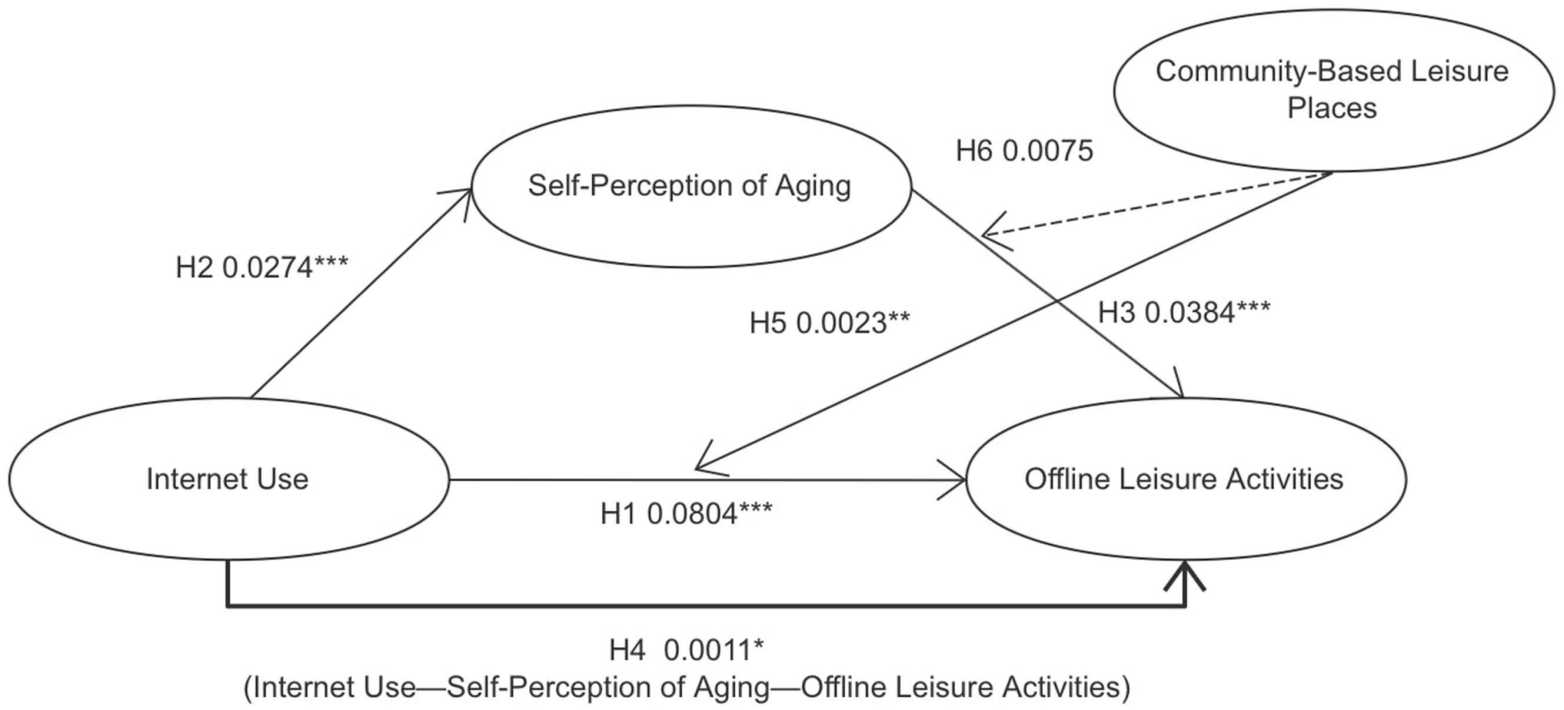
The results of moderated mediation model.

As illustrated in Figure 4, there is a significant positive correlation between Internet use and offline leisure activities (*β* = 0.0804, *p* < 0.0001). This provides support for H1. The results indicated a significant positive correlation between Internet use and self-perception of aging (*β* = 0.0274, *p* < 0.0001), thereby supporting H2. Self-perception of aging demonstrated a significant positive correlation with offline leisure activities (*β* = 0.0384, *p* < 0.0001), thereby supporting hypothesis H3. Self-perception of aging played a significant, positive mediating role (*β* = 0.0011, *p* < 0.05) between Internet use and offline leisure activities, thereby supporting H4. Community-based leisure places exerted a significant positive moderating effect between Internet use and offline leisure activities (*β* = 2.8942, *p* < 0.01), which supported H5. However, the moderating effect of community-based leisure places between self-perception of aging and offline leisure activities did not reach statistical significance (*β* = −1.4852, *p* > 0.05). Consequently, H6 was not supported (Figure 5).

This structural engineering model predicted the impact of Internet use by older adults on offline leisure activities, with Self-perception of aging as the mediating variable and Community-based leisure places as the moderating variable. Statistics are standardized regression coefficients. A bold solid line represents a significant indirect path; solid lines represent significant direct paths; a dotted line represents a nonsignificant relation. ^*^p < 0.01. ^**^p < 0.001.

## 5 Discussion

In order to provide an important theoretical and factual basis for the solution of China’s aging problem, CLASS has adopted a multi-stage stratified probability sampling method to regularly and systematically collect data on the social and economic background of China’s older adult population (88–91). Our research is based on data from the screened CLASS 2020, which covers a total valid sample of 8,180 Chinese older people, and the results of the study can effectively represent the overall situation of Chinese older people (92). This study explores the relationship between online and offline leisure activities of Chinese older adults in the Internet era by constructing an SEM model. Our findings corroborate the conclusions of analogous gerontological studies conducted elsewhere in the world. Furthermore, we offer pertinent recommendations for the advancement of an aging society in China and internationally. In addition, we paid particular attention to the mediating role of self-perception of aging as a subjective psychological factor and the moderating role of community-based leisure places as an objective environmental factor. The theoretical and practical results are as follows.

### 5.1 Theoretical aspects

Firstly, the findings supported H1 among Chinese seniors, active Internet use is significantly and positively associated with active offline leisure activities. This result is consistent with the findings of a study conducted in Finland by Näsi et al. which demonstrated a significant positive correlation between Internet use and leisure activities (offline), and that active aging lifestyles frequently involve online access (40). Our research findings are also in accordance with the findings of Mano and Rosenberg’s study of online-offline interactions (37). Among the older population, the Internet has strengthened rather than weakened offline social connections, and online channels facilitate the creation of and participation in social space (37). Both online socialization and offline leisure activities create and maintain close connections between people with similar interests, and in this era, people are in line with young and middle-aged populations (92, 93). We conclude that online socialization removes the social barriers caused by spatial and temporal separation, eases communication costs, and broadens communication channels, while offline leisure activities expand the membership of online socialization. The conclusion that activities of diverse types (online and offline) can reinforce and supplement each other is equally applicable to the Chinese older age group.

Secondly, the results of this study provide support for H2, H3, and H4. The positive mediating role of self-perception of aging was empirically supported. With the use of the Internet, the activities of older adults are becoming more and more similar to those of young and middle-aged adults, which facilitates the maintenance of a more positive, youthful aging perception (40). This positive subjective attitude in turn feeds positively back into real-world activities, helping to maintain more active offline leisure activities. Consistent with younger people, the use of the Internet by older people can be effective in improving psychological well-being (94). Active theory of aging is also used in the Internet arena (64). The Internet has become a medium for social interaction and connection between people, providing various social roles and social support for the older adults (11, 58, 64). The social roles and support provided by the Internet tend to lead silver surfers to have a more positive aging perception, which is significantly and positively correlated with being more socially active.

Subsequently, we propose two hypotheses of moderating effects. The positive moderating effect of community-based leisure places as a community environment on the relationship between Internet use and offline leisure activities was demonstrated in the moderating effects test, and H5 was thus supported. The establishment of H5 confirms the theory of Environmental Gerontology that the setting of community recreational facilities and places strengthens the positive relationship between online and offline activities for older adults. In a system of nested individual subjective factors and objective environment, the environment plays a pivotal role in encouraging individual participation in leisure activities (54, 55). An inclusive community environment is effective in enhancing individual community integration (59). The findings of this empirical study with Chinese older adult individuals align with those of the study conducted by Śniadek and Zajadacz in Poland (78). Theories pertinent to Environmental Gerontology are likewise applicable in the Chinese context. While personal factors are an important part of maintaining long-term, well-being for seniors, environmental factors also play a significant role (54). The moderating effect of community-based leisure places on the relationship between self-perception of aging and offline leisure activities was not found to be significant, and thus, H6 was not supported. The CLASS 2020 questionnaire survey was conducted during the COVID-19 pandemic when China maintained a harsh policy that prohibited people from gathering in public places and kept people isolated at home for long periods of time (95, 96). Many of China’s epidemic prevention efforts are community-based and have a long-term, profound impact on people’s lives (97). There is a possibility that the moderating effect of community-based leisure places, as an environmental factor, could be squeezed by public health events and immunization policies, and thus was not significant in this study.

Finally, the frequency description of the “Internet use” variable reveals that respondents exhibit polarization in their usage patterns. This polarization is often defined as the “digital divide,” which refers to the gaps and inequalities in access to information (98, 99). With 25% of older people being frequent Internet users, it can be said that Internet use has become an integral part of their lives. However, 75% of the respondents are not accustomed to using the Internet at all, which means that the vast majority of China’s older adults are likely to suffer from the injustices of the “digital divide” (100, 101).

### 5.2 Practical aspects

The Internet can be considered a positive force in actively addressing the issues of an aging society. To fully utilize the positive functions of the Internet in aging issues, the government, universities, occupational therapists, and the community must work together. The government should make the Internet an important element of the public service infrastructure and build an online-offline interaction smart older adult care system (102, 103). In this process, the government should emphasize personal privacy and data security. It is necessary for the government to improve relevant laws and policies to provide institutional safeguards to protect older users from online fraud and data leakage (104). In the courses of occupational therapists, colleges should emphasize the potential of the Internet to foster age-friendly, Internet-based occupational therapy programs, such as teletherapy courses, online cognitive training, and virtual support groups (17, 105). In the geriatric rehabilitation process, occupational therapists need to consciously guide older adults to use the Internet to provide health-related services through telehealth (105). The community should focus on infrastructure development and building age-appropriate Internet education centers (106, 107). In addition, the community can coordinate with software engineers to develop age-friendly software systems with features such as font size enlargement, one-button distress, and voice announcements (108). There is every reason to believe that a multi-participant approach can enhance the benefits of Internet use for older adults.

Active self-perception of aging can have a positive impact on the health, longevity, and functioning of older adults (82, 109). Shaping the concept of “active aging” requires the participation of multiple actors, including the government, educational institutions, and occupational therapists. The government should guide society to shape an active attitude toward aging through policies, to dissipate people’s fear and prejudice against getting old (110). The government should also emphasize the protection and promotion of public health and implement a healthcare system that covers the entire population, especially preventive health services for the older adults (110). In addition, the government should encourage family doctors and community service centers to pay special attention to the psychological state of the older adults. Educational institutions are key players in shaping society’s perception of active aging. On the one hand, intergenerational education programs can be introduced in compulsory education to enhance the younger generation’s empathy and understanding of the older adults (111). On the other hand, community colleges and universities should provide lifelong learning opportunities for older persons, such as hobby courses or vocational skills training, to help them continue to adapt to modern society (8). Occupational therapists should focus on shaping the active perception of aging during activity interventions with older adults (112). For example, by changing the physical conditions, behavioral styles, and psychological states of the older adults, the social mobility of the older adults is enhanced, thus increasing their confidence in life. Finally, there is a need to promote the dissemination of active perception of aging by the media, social organizations, and enterprises, to create an inclusive attitude toward older persons in society.

The community environment has a significant positive moderating effect on older people’s activities. An age-friendly community environment requires the involvement of government, community, and occupational therapists. The government can improve the community environment through policy guidance and financial support. In our opinion, the government should allocate funds to the community for infrastructure improvements, which provide walking paths, wheelchair ramps, and well-maintained parks for older persons (113). At the same time, the government needs to foster community organizations that care for the older adults, create projects focusing on the older adults, and actively guide offline leisure activities for the older adults (113). It is recommended that the community work with the government to establish a greater number and variety of recreational areas for the older adults within a more reasonable distance (58, 62, 113). In addition, the community should provide volunteer services and guidance for older persons and organize community activities such as cultural festivals and group exercises, thereby building an age-friendly community humanities environment (114). It is recommended that occupational therapists assess and intervene in community settings (59, 115). Furthermore, occupational therapists should actively communicate with the government and the community to make timely adjustments to the infrastructure for the older adults. We believe that through the joint efforts of all parties, we can effectively improve the living environment of the older adults and build a safe and friendly community for the older adults.

It is also crucial to underscore that nearly 75% of the older respondents are currently non-Internet users. Consequently, the digital divide is an issue that affects the majority of the Chinese older adult population. The phenomenon of the “gray digital divide” (101) in relation to older persons can be observed in a multitude of countries across the globe (116, 117). This situation was exacerbated during the COVID-19 period when a very large number of older adult people in China were isolated from their daily lives due to their lack of access to the Internet. In response, government-led digital inclusion policies and digital education programs are imperative (118, 119). On the one hand, governments should strengthen Internet infrastructure in remote rural areas and provide free or subsidized Internet devices for the older adults. On the other hand, the Government should take a number of specific measures to provide targeted training for the older adults who are caught in the “digital divide.” Include “Internet use through smartphones” in the permanent non-profit courses offered by the local university for the older adults and increase the frequency of the courses (guarantee a frequency of at least one course per week). Mobilize social forces to include “teaching the older adults how to use smartphones” as a service component of volunteer teams. Encourage telecommunication companies to provide public service teaching for the older adults and send relevant personnel to provide one-on-one personalized services. At the same time, to ensure the older adults who do not use the Internet are treated fairly, the government and society should pay attention to the maintenance and provision of offline public service facilities.

## 6 Limitations

Despite the theoretical and practical contributions, this study still has some limitations. Firstly, the current study is a cross-sectional study, which may limit the generalizability of the item results over a longitudinal time period. Of particular note, the data for this study were collected from the COVID-19 pandemic period. During that period, the Chinese government maintained a strict policy that curtailed the activities of its citizens. Therefore, this study needs to be followed up with further exploration from a longitudinal timeline perspective, especially pre-epidemic, intra-epidemic, and post-epidemic. Secondly, the study did not divide the respondents, and subsequent studies could investigate the situation of Internet use among older adults in economic and geographic distinctions by dividing them into East, Central, and West regions or by dividing them into rural and urban areas. In addition, this study explored the mediating role of self-perception of aging. Future research could also include factors such as the subjective health of older adults and the emotional support provided by family members to older adults as mediating variables. Finally, this paper uses community-based leisure places as a proxy for the community environment. Future models could use other moderating variables such as the activity organizations in the community, and older adult services available in the community.

Given the correlational nature of this study, causal inferences should be made with caution.

## 7 Conclusion

This paper pays special attention to the relationship between the frequency of Internet use and offline leisure activities among the older adults in the Chinese scenario, which is mutually supported and corroborated with similar studies in other parts of the world. Moreover, it offers empirical evidence to support the implementation of active aging policies and environmental gerontology theories in the context of the Internet age. It is notable that this study demonstrated the mediating role of self-perception of aging and the positive moderating role of community-based leisure places. With an active and conscious lifestyle intervention, the Internet can be a beneficial tool for the physical and mental health of the older adults and for enriching their lives. Nevertheless, it is imperative that conscious efforts be made to popularize the digital skills of the older adults and to construct an age-friendly Internet. Otherwise, older people are likely to be trapped in the disadvantageous situation of the digital divide. In addition, it is crucial to acknowledge that a supportive and age-friendly environment is vital for successful and active aging.

In sum, the findings are both academically and practically significant. Since population aging is a triumph of advances in medical technology and public health, there is reason to believe that the antidote to the problem of aging is also embedded in the process of technological and social development. The promotion of the physical and mental health of older persons is of benefit to the common good of humanity. The objective of this study is to prompt academics and public policymakers to consider the needs of an aging society from an integrated perspective encompassing technology, social perception, and the social environment.

## Data Availability

The data underlying this article were provided by the Institute of Gerontology, Renmin University of China, by permission. Data will be shared on request to the corresponding author with permission of the Institute of Gerontology, Renmin University of China. E-mail of the third party: class_ruc@163.com.

## Footnotes

^1^As a consequence of COVID-19, CLASS 2020 was made available to the public in December 2023. It is currently the most recent tracking survey on the aging society in China.

^2^Law of the People’s Republic of China on the Protection of the Rights and Interests of the older adult, Article 3 of Chapter 1: older adult persons are citizens over 60 years of age. Therefore, this paper defines the age of the older adult in China as 60 years and above. This is in contrast to some countries and regions where the age of 65 and above is defined as old age.

^3^class_ruc@163.com.

^4^Combined with comprehensive indicators of geography and socio-economic development, China is divided into three regions: East, Central and West. In this paper, the eastern part of China consists of 11 provinces (cities): Tianjin, Liaoning, Hebei, Beijing, Shandong, Jiangsu, Shanghai, Zhejiang, Fujian, Guangdong and Hainan. The central includes “Heilongjiang, Inner Mongolia, Jilin, Shanxi, Anhui, Jiangxi, Henan, Hubei, Hunan” nine provinces (autonomous regions). The western part of China includes 11 provinces (autonomous regions and cities): Shaanxi, Gansu, Ningxia, Qinghai, Xinjiang, Guizhou, Tibet, Sichuan, Chongqing, Yunnan and Guangxi.
